# Effectiveness of different booster vaccine combinations against SARS-CoV-2 during a six-month follow-up in Mexico and Argentina

**DOI:** 10.3389/fimmu.2024.1403784

**Published:** 2024-05-14

**Authors:** Arnulfo Garza-Silva, Diego Rivera-Salinas, Andrea Rivera-Cavazos, Iván Francisco Fernández-Chau, Andrea Belinda Cepeda-Medina, Devany Paola Morales-Rodríguez, Irene Antonieta Barco-Flores, Miguel Ángel Sanz-Sánchez, Cecilia Acciardi, Graciela Paez-Bo, Mauro M. Teixeira, Elena Azzolini, Chiara Pozzi, Maria Rescigno, Maria Elena Romero-Ibarguengoitia

**Affiliations:** ^1^ Research Department, Hospital Clínica Nova de Monterrey, San Nicolás de los Garza, Nuevo León, Mexico; ^2^ Vicerrectoría de Ciencias de la Salud, Escuela de Medicina, Universidad de Monterrey, San Pedro Garza García, Mexico; ^3^ Health Secretary, Unidad Hospitalaria San José, Campana, Argentina; ^4^ Laboratory Department, Hospital Interzonal General de Agudos San Felipe, San Nicolás de los Arroyos, Argentina; ^5^ Biochemistry and Immunology Department, Instituto Ciencias Biologicas (ICB), Universidad Federal de Minas Gerais, Belo Horizonte, Brazil; ^6^ Instituti di Ricovero e Cura a Carattere Scientifico (IRCCS) Humanitas Research Hospital, Milan, Italy; ^7^ Department of Biomedical Sciences, Humanitas University, Milan, Italy

**Keywords:** vaccination, COVID-19, SARS-CoV-2, antibodies, booster dose, re-vaccination

## Abstract

**Introduction:**

Given the limited number of patients in Latin America who have received a booster dose against the COVID-19, it remains crucial to comprehend the effectiveness of different vaccine combinations as boosters in real-world scenarios. This study aimed to assess the real-life efficacy of seven different vaccine schemes against COVID-19, including BNT162b2, ChAdOx1-S, Gam-COVID-Vac, and CoronaVac as primary schemes with either BNT162b2 or ChAdOx1-S as booster vaccines.

**Methods:**

In this multicentric longitudinal observational study, participants from Mexico and Argentina were followed for infection and SARS-CoV-2 Spike 1–2 IgG antibodies during their primary vaccination course and for 185 days after the booster dose.

**Results:**

A total of 491 patients were included, and the booster dose led to an overall increase in the humoral response for all groups. Patients who received BNT162b2 exhibited the highest antibody levels after the third dose, while those with primary Gam-COVID-Vac maintained a higher level of antibodies after six months. Infection both before vaccination and after the booster dose, and Gam-COVIDVac + BNT162b2 combination correlated with higher antibody titers.

**Discussion:**

The sole predictor of infection in the six-month follow-up was a prior COVID-19 infection before the vaccination scheme, which decreased the risk of infection, and all booster vaccine combinations conveyed the same amount of protection.

## 1 Introduction

The pandemic caused by the Severe Acute Respiratory Syndrome Coronavirus 2 (SARS-CoV-2) had a profound impact on global health, and an especially greater impact in countries with limited healthcare infrastructure (1, 2). Throughout the pandemic, Latin America experienced high mortality rates and a lack of access to adequate health resources, including limited access to vaccines which took the most crucial role in mitigating the spread of disease and its severity (3–5). As for December 2023, Brazil is the Latin American country most affected by the COVID-19 pandemic with a report of 37.5 million cumulative cases, followed by Argentina (10 million) and Mexico (7.7 million) (6). The significant population size and constrained healthcare infrastructure limited these countries to be leaders in vaccination efforts across the region. Cuba and Chile, on the other hand, stood out by administering 300 vaccines per 100 inhabitants as of November 2022, while the former three nations fell behind with 175-240 vaccines administered per 100 inhabitants. This indicates that, as of November 2022, approximately half of the population in these countries had not yet received the booster vaccine dose. The most recent data from Argentina reports 256 vaccines per 100 inhabitants (7).

Latin America adopted a diverse range of vaccines for its population, administering them promptly upon availability despite disparities in acquisition (3). This approach led to a population with exposure to various vaccine types. Concurrently, the concept of booster vaccinations gained attention as a potential strategy to enhance, optimize, and sustain immunity against SARS-CoV-2 (8). This approach involves administering a booster dose using either the same vaccine given for the initial doses (homologous booster) or a different type (heterologous booster). Due to the observed decline in antibody levels over time and the promising results of booster vaccines, this strategy was quickly embraced by the Strategic Advisory Group of Experts on Immunization (SAGE) from the World Health Organization (WHO), who recommended the use of booster vaccines to restore and extend the protective effect in individuals who had at least one vaccine (9).

Previous existing research highlights the importance of booster doses in addition to standard primary vaccination regimens such as BNT162b2, mRNA-1273, or ChAdOx1-S (10–12). Studies on both heterologous and homologous booster vaccinations have yielded promising results, suggesting that a heterologous booster could elicit more robust immune responses and potentially addressing vaccine hesitancy in certain populations (13, 14). To our current knowledge, there is a scarcity of Real-World studies in Latin America that comprehensively evaluate and compare various vaccine combinations, including those with limited research such as Gam-COVID-Vac. We believe incorporating data from these less-explored combinations could enhance our understanding of heterologous booster efficacy (15–17).

The aim of this study is to analyze the humoral response, efficacy, and reactogenicity of booster combinations for diverse primary vaccine schemes in a real-world, multicentric setting with patients from Argentina and Mexico, two of the most affected countries by COVID-19 across Latin America. This study will provide valuable insights into the feasibility and impact of homologous and heterologous booster vaccination in this region, as well as contribute to our growing knowledge on heterologous booster vaccination strategies and their distinct advantages.

## 2 Materials and methods

The following is a multicentric observational longitudinal study, composed of subjects from two of the most affected countries by the COVID-19 pandemic in Latin America, Argentina and Mexico, in which volunteers from two different hospital centers (Hospital Municipal San Jose, Hospital Interzonal de Agudos San Felipe and Hospital Clinica Nova) who had received a complete scheme of the approved vaccines and a booster, either homologous or heterologous, were followed for 185 days after the last dose.

The design of the study followed the Strengthening the Reporting of Observational studies in Epidemiology (STROBE) guidelines and was approved by each local institutional Review Board, and conducted in accordance to the Code of Ethics of the World Medical Association (Declaration of Helsinki) for experiments involving humans (18, 19). In reference to selection criteria, we included volunteered patients of any age, both genders, who gave consent to participate, and were planning on completing the vaccination scheme, who agreed to be followed through the studies duration. We excluded patients who received a prior SARS-CoV-2 before starting the study and those who received an heterologous combination scheme (meaning that they completed the common 2 dose vaccines with different vaccines). We eliminated patients lost during the follow-up, and those that did not receive booster doses, either heterologous or homologous.

Since this was a real-world study, the availability of vaccines was defined by the public health systems of Argentina and Mexico, respectively at the time patients were enrolled. Subjects enrolled in the study received 3 doses, 2 from a specific vaccination scheme and a booster dose which could have been either from the same vaccine producer or a different one. Subjects received the doses during 2021-2022 and were exposed to different SARS-CoV-2 variants, including Alpha, Beta, Gamma, Delta, Epsilon, Eta and Omicron strains.

**Figure 1:**
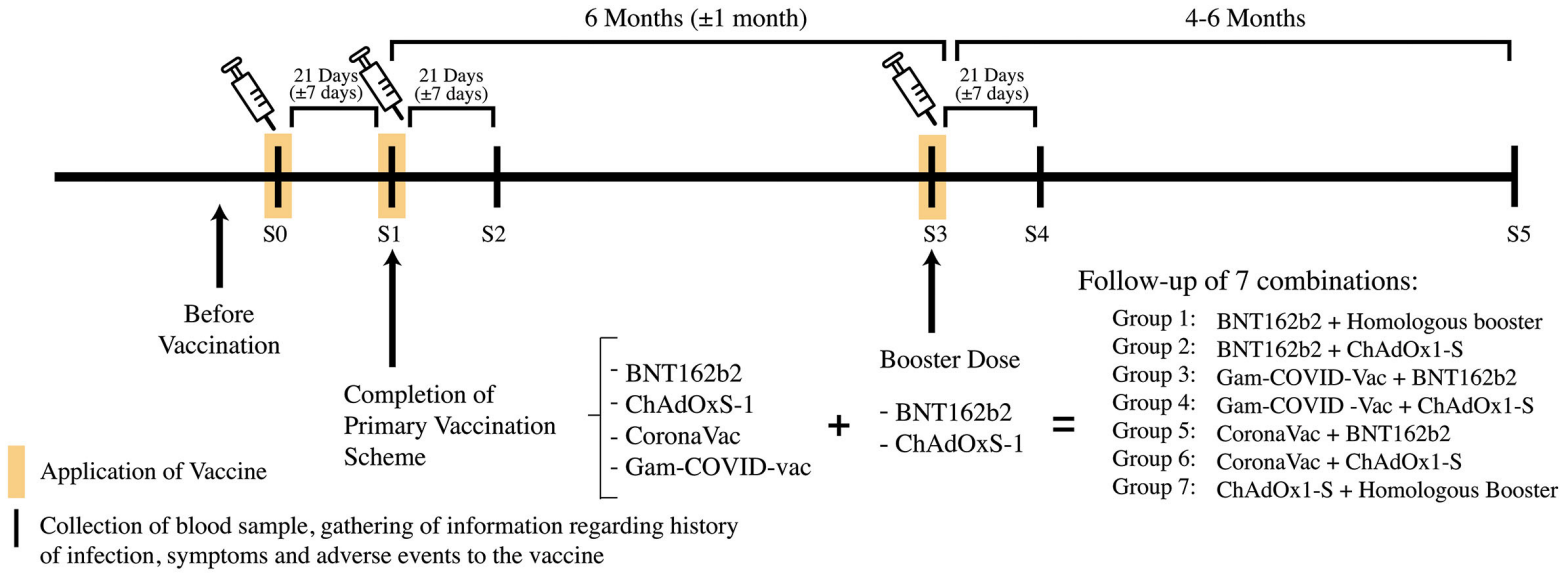
Methodology regarding the acquisition of samples and follow-up. In the timeline the black lines represent the time were blood samples or information regarding infection and vaccination were gathered, the yellow frames represent application of vaccine. A total of 7 vaccine combinations were followed during 4-6 months after the application of the booster dose.

Patients were invited to participate before receiving the first dose where the research team explained thoroughly the project. Those interested and qualified, after the selection criteria, were given a consent form which they signed upon agreement to participate. A plasma baseline sample (S0) was taken at this point in order to measure serum SARS-CoV-2 Spike 1-2 IgG antibodies. The first (S1) and second (S2) samples were taken 21 (+/- 7) days after each vaccine application, then preboost antibodies (S3) were taken 6 (+/- 1) months after the completion of the scheme and just before the application of the booster dose, a sample was taken 21 (+/- 7) days after the booster dose (S4) and a last sample was taken 4-6 months after the booster dose (S5). As it shows in **Figure 1**.

In each follow-up sample, the participants were given questionnaires regarding medical history, vaccination scheme and side effects, and SARS-CoV-2 infection history (including symptoms and management of the disease) before and during the follow-up. The preboost (S3) and postboost (S5) questionnaires only involved questions about the SARS-CoV-2 infection history.

The measurements for SARS-CoV-2 Spike 1-2 IgG antibodies were made in a quantitative manner, using the DiaSorin’s chemiluminescence immunoassay (CLIA). This assay had a sensitivity of 97.4% (95% CI, 86.8-99.5) and a specificity of 98.5% (95 CI, 97.5-99.2). Interpretation of the results was as follows: values <12.0 AU/mL were considered negative results; a result between 12.0-15.0 AU/mL was considered indeterminate; and values >15 AU/mL were considered a positive result (20). The same kit has been used in previous studies (21–24).

The present study conducted analysis on variables regarding sex, age, medical history of the patients (i.e. type 2 diabetes mellitus, hypertension, and other diseases), the history of confirmed SARS-CoV-2 infection (either by nasal swab and PCR or viral protein antigen detection), medical management in case of disease (ambulatory or hospitalization) and need of supplementary oxygen. We included the analysis of the antibody titers as previously described. Efficacy of vaccination was measured through symptomatic infection and humoral response at different time points.

### 2.1 Statistical analysis

Before analyzing the data, researchers assessed its quality control and anonymity. Normality was evaluated through either the Shapiro-Wilk or Kolmogorov-Smirnov tests. Descriptive analysis for data with a normal distribution was reported using the mean and standard deviation, while data that couldn’t be normalized were reported using the median and interquartile range. Categorical data were presented as frequencies and percentages. The chi-square and fisher’s exact tests were employed for categorical variables related to medical history, COVID-19 symptoms and adverse reactions to the vaccines. Antibodies were analyzed using the Kruskal-Wallis test between groups of vaccines and the Friedman test. Additionally, Mann-Whitney tests were used for intergroup analysis over time. A linear regression model was utilized to predict antibody titers at the 6-month (+/− 1 month) follow-up after the booster dose. The analysis included only subjects with known antibody titers after the 3rd vaccine dose and at the 6-month follow-up. The model incorporated the following covariates: sex, age, vaccine group combination (with a homologous booster of BNT162b2 as a reference), previous SARS-CoV-2 infection (before the 1st dose), and SARS-CoV-2 infection after the booster dose. A survival curve analysis and Cox proportional hazard model were employed to identify predictor factors for SARS-CoV-2 infection after the booster dose. The model included the following covariates: sex, age, history of diabetes mellitus type 2, history of hypertension, vaccine group combination (with a homologous booster of BNT162b2 as a reference), and previous SARS-CoV-2 infection (before the 1st dose). Additionally, only individuals with no missing values were included in the model. The event considered was the first SARS-CoV-2 infection after the booster dose. The time-to-event was defined as the days between the booster dose and the occurrence of SARS-CoV-2 infection. Individuals with no SARS-CoV-2 infections after the booster dose were treated as censored cases, with the time-to-censoring being the days elapsed between the booster dose and the follow-up date. The endpoint for the Cox model was set at 185 days, which was the last day the patients were followed. A p-value less than 0.05 was considered statistically significant. Missing random values were analyzed through complete case analysis. The statistical programs used were SPSS v.27 and R v. 4.0.3.

## 3 Results

A total of 491 patients were analyzed in this multicentric study, 413 of them from Mexico and 78 from Argentina. The median (IQR) age was 57 (23) years old and 252 (51.3%) of the patients were women. A total of 7 groups of different vaccine combinations were explored in this study. Patients with a completed CoronaVac scheme and a third dose booster shot with ChAdOx1-S were the most prominent group with 193 (39.3%) patients. The next biggest group were the patients with a complete scheme and booster with ChAdOx1-S in 141 (28.7%), followed by the complete Sputnik V scheme with an ChAdOx1-S booster in 49 (10%), complete scheme and booster with BNT162b2 in 42 (8.6%), complete Sputnik V scheme plus a booster with BNT162b2 in 29 (5.9%), complete scheme with BNT162b2 and a ChAdOx1-S booster, and lastly a group of complete CoronaVac scheme plus a booster of BNT162b2 in 10 (2%) patients. The most prevalent comorbidities in patients were hypertension with 161 (32.8%) patients, obesity in 143 (29.1%) and dyslipidemia in 105 (21.4%). The medical history of the distinct groups of heterologous vaccination schemes can be observed in **Table 1**.

**Table 1:** Medical history divided by heterologous vaccination scheme.

Variables	BNT162b2 + Homologous booster n= 42 (8.6%)	BNT162b2 + ChAdOx1-S n= 27 (5.5%)	Gam-COVID-Vac + BNT162b2 n= 29 (5.9%)	Gam-COVID -Vac + ChAdOx1-S n= 49 (10%)	CoronaVac + BNT162b2 n= 10 (2%)	CoronaVac + ChAdOx1-S n= 193 (39.3%)	ChAdOx1-S + Homologous Booster n= 141 (28.7%)	Total vaccines n= 491	p-value
Female	27 (64.3)	9 (33.3)	16 (55.2)	29 (59.2)	5 (50)	96 (49.7)	70 (49.6)	252 (51.3)	0.233
Age ^a^	44 (13)	34 (8)	66 (9)	72 (22)	48.5 (19	51 (11)	71 (15)	57 (23)	<0.001
Age ≥60	2 (4.8)	0 (0)	28 (96.6)	47 (95.9)	2 (20)	0 (0)	129 (91.5)	208 (42.4)	<0.001
Hypertension	7 (16.7)	1 (3.7)	11 (37.9)	27 (55.1)	3 (30)	45 (23.3)	67 (47.5)	161 (32.8)	<0.001
Obesity	6 (14.3)	7 (25.9)	5 (17.2)	8 (16.3)	2 (20)	62 (32.1)	53 (37.6)	143 (29.1)	0.011
Dyslipidemia	3 (7.1)	2 (7.4)	9 (31.0)	20 (40.8)	4 (40)	27 (13.9)	40 (28.4)	105 (21.4)	<0.001
Type 2 Diabetes Mellitus	2 (4.8)	0 (0)	1 (3.4)	14 (28.6)	3 (30)	25 (12.9)	47 (33.3)	92 (18.7)	<0.001
Asthma	0 (0)	0 (0)	0 (0)	0 (0)	1 (10)	5 (2.6)	2 (1.4)	8 (1.6)	0.241
COPD	0 (0)	0 (0)	1 (3.4)	1 (2.0)	0 (0)	0 (0)	2 (1.4)	4 (0.8)	0.394
Smoking	5 (11.9)	6 (22.2)	4 (13.8)	4 (8.2)	0 (0)	21 (10.9)	6 (4.2)	46 (9.4)	0.057
Kidney disease	1 (2.4)	0 (0)	0 (0)	0 (0)	0 (0)	3 (1.5)	4 (2.8)	8 (1.6)	0.765
Active neoplasia	0 (0)	0 (0)	0 (0)	0 (0)	0 (0)	0 (0)	3 (2.1)	3 (0.6)	0.278
Previous neoplasia	0 (0)	0 (0)	0 (0)	0 (0)	0 (0)	3 (1.5)	11 (7.8)	14 (2.8)	0.006
Atrial fibrillation	0 (0)	0 (0)	3 (10.3)	4 (8.2)	1 (10)	2 (1.0)	6 (4.2)	16 (3.2)	0.016
Chronic heart failure	0 (0)	0 (0)	5 (17.2)	8 (16.3)	0 (0)	0 (0)	5 (3.5)	18 (3.7)	<0.001
Previous coronary artery disease	1 (2.4)	0 (0)	3 (10.3)	9 (18.4)	0 (0)	2 (1.0)	5 (3.5)	20 (4.1)	<0.001
Previous stroke	0 (0)	0 (0)	0 (0)	0 (0)	0 (0)	1 (0.5)	4 (2.8)	5 (1.0)	0.347
Hepatic steatosis	0 (0)	0 (0)	2 (6.9)	1 (2.0)	0 (0)	9 (4.7)	5 (3.5)	17 (3.5)	0.545
Cirrhosis	0 (0)	0 (0)	0 (0)	0 (0)	0 (0)	1 (0.5)	2 (1.4)	3 (0.6)	0.868
Other liver diseases	0 (0)	0 (0)	1 (3.4)	2 (4.1)	1 10	3 (1.5)	2 (1.4)	9 (1.8)	0.32
Rheumatoid arthritis or other rheumatological diseases	0 (0)	0 (0)	1 (3.4)	8 (16.3)	0 (0)	4 (2.1)	14 (9.9)	27 (5.5)	<0.001
Other immune system diseases such as thyroiditis or psoriasis	3 (7.1)	1 (3.7)	0 (0)	0 (0)	0 (0)	13 (6.7)	15 (10.6)	32 (6.5)	0.107
Treatment with immunosuppressants	2 (4.8)	0 (0)	1 (3.4)	1 (2.0)	0 (0)	1 (0.5)	1 (0.7)	6 (1.2)	0.287
Gout	0 (0)	0 (0)	3 (10.3)	3 (6.1)	0 (0)	7 (3.6)	11 (7.8)	24 (4.9)	0.157
Surgery within the last year under general anesthesia	2 (4.8)	1 (3.7)	1 (3.4)	1 (2.0)	1 (10)	8 (4.1)	13 (9.2)	27 (5.5)	0.38

Data are presented as frequencies and percentages. Chi square test was used for comparison. A p-value < 0.05 was considered statistically significant. ^a^ This variable is presented in median (IQR) and the p-value calculated with the Kruskal-Wallis test.

### 3.1 Adverse events following immunization

The adverse events following immunization (AEFI) were assessed following each of the three vaccine doses. A noticeable reduction in AEFI occurred after the second dose, declining from 226 (46%) cases after the first dose to 156 (31.8%) and 169 (34.4%) following the second and booster doses, respectively. Analysis of AEFI incidence after the booster dose revealed the highest rates among patients receiving CoronaVac + BNT162b2 and CoronaVac + ChAdOx1-S [7 (87.5%) and 100 (71.4%), respectively]. In contrast, primary Gam-COVID-Vac with BNT162b2 and primary BNT162b2 + ChAdOx1-S exhibited the lowest adverse event rates [9 (18.4%) and 7 (25.9%), respectively], p<0.001.

Incidence of AEFIs was notably elevated in the CoronaVac + BNT162b2 and Coro-naVac + ChAdOx1-S groups [7 (87.5%) and 100 (71.4%), respectively]. Following the booster dose, an increase in the number of moderate reactions was observed in com-parison to the first and second doses [46 (27.2%) vs. 24 (10.6%) and 11 (7.1%), respectively]. This escalation was primarily attributed to the CoronaVac + ChAdOx1-S and BNT162b2 + ChAdOx1-S groups [37 (37%) and 3 (42.9%), respectively]. Conversely, patients from the groups with the primary Gam-COVID-Vac and BNT162b2 or ChAdOx1-S exhibited only very mild reactions.

Severe reactions were infrequent, with only four cases identified. Two cases were associated with CoronaVac + ChAdOx1-S, while the other two occurred in the BNT162b2 + Homologous booster and ChAdOx1-S + Homologous booster groups. Details from the primary schemes are available in **Table 2**.

**Table 2:** Adverse events following vaccination categorized by severity and separated by groups of heterologous vaccination schemes.

Variables		BNT162b2 + Homologous booster n= 42 (8.6%)	BNT162b2 + ChAdOx1-S n= 27 (5.5%)	Gam-COVID -Vac + BNT162b2 n= 29 (5.9%)	Gam-COVID -Vac + ChAdOx1-S n= 49 (10%)	CoronaVac + BNT162b2 n= 10 (2%)	CoronaVac + ChAdOx1-S n= 193 (39.3%)	ChAdOx1-S + Homologous Booster n= 141 (28.7%)	Total vaccines n= 491	p-value
Adverse reaction to the first vaccine		32 (76.2)	21 (77.8)	13 (44.8)	13 (26.5)	6 (60)	78 (41.3)	63 (45)	226 (46.0)	<0.001
Reactions Severity	Very Mild	14 (43.8)	14 (66.7)	4 (30.8)	4 (30.8)	4 (66.7)	45 (57.7)	34 (57.6)	119 (52.7)	0.016
	Mild	11 (34.4)	7 (33.3)	9 (69.2)	7 (53.8)	2 (33.3)	27 (34.6)	14 (23.7)	77 (34.1)	
	Moderate	7 (21.9)	0 (0)	0 (0)	1 (7.7)	0 (0)	5 (6.4)	11 (18.6)	24 (10.6)	
	Severe	0 (0)	0 (0)	0 (0)	1 (7.7)	0 (0)	1 (1.3)	0 (0)	2 (0.9)	
Adverse reaction to the second vaccine		25 (62.5)	16 (66.7)	9 (31.0)	11 (22.4)	4 (40)	66 (36.1)	25 (18.6)	156 (31.8)	<0.001
Reactions Severity	Very Mild	15 (60)	8 (50)	1 (11.1)	3 (27.3)	3 (75)	42 (63.6)	11 (44)	83 (53.2)	0.052
	Mild	6 (24)	7 (43.8)	8 (88.9)	8 (72.7)	1 (25)	20 (30.3)	11 (44)	61 (39.1)	
	Moderate	4 (16)	1 (6.3)	0 (0)	0 (0)	0 (0)	4 (6.1)	2 (8)	11 (7.1)	
	Severe	0 (0)	0 (0)	0 (0)	0 (0)	0 (0)	0 (0)	1 (4)	1 (0.6)	
Adverse reaction to the booster vaccine		20 (57.1)	7 (25.9)	12 (41.4)	9 (18.4)	7 (87.5)	100 (71.4)	14 (31.1)	169 (34.4)	<0.001
Reactions Severity	Very Mild	6 (30)	3 (42.9)	12 (100)	9 (100)	0 (0)	33 (33)	10 (71.4)	73 (43.2)	<0.001
	Mild	8 (40)	1 (14.3)	0 (0)	0 (0)	5 (71.4)	28 (28)	3 (21.4)	45 (26.6)	
	Moderate	5 (25)	3 (42.9)	0 (0)	0 (0)	2 (28.6)	37 (37)	0 (0)	46 (27.2)	
	Severe	1 (5)	0 (0)	0 (0)	0 (0)	0 (0)	2 (2)	1 (7.1)	4 (2.4)	

Data are presented as frequencies and percentages. Chi square test was used for comparison. A p-value < 0.05 was considered statistically significant.

### 3.2 SARS-CoV-2 spike 1-2 IgG antibodies during the follow-up

The SARS-CoV-2 Spike 1-2 IgG antibody titers were analyzed through the different vaccination scheme groups. The patients were divided from each group into three groups upon history of infection (COVID-19): (1) patients with negative history of infection (naive group), (2) positive history of infection before vaccination, and (3) new cases of infection through the follow-up. It is shown that individuals from group 1 tend to have less antibodies as compared to the other groups. **Table 3** shows the median (IQR) S1/S2 IgG by group and time-point of measurement.

**Table 3:** Median (IQR) IgG SARS-CoV-2 S1-S2 antibody titers by vaccination scheme and SARS-CoV-2 infection history.

SARS-CoV-2 Infection History	Total vaccines n= 491 (%)	BNT162b2 + Homologous Booster n= 42 (8.6%)	BNT162b2 + ChAdOx1-S n= 27 (5.5%)	Gam-COVID-Vac + BNT162b2 n= 29 (5.9%)	Gam-COVID-Vac + ChAdOx1-S n= 49 (10%)	CoronaVac + BNT162b2 n= 10 (2%)	CoronaVac + ChAdOx1-S n= 193 (39.3%)	ChAdOx1-S + Homologous Booster n= 141 (28.7%)	p-value
Before vaccination									
Negative	3.8 (0) (n=340)	3.8 (0) (n=22)	3.8 (0) (n=19)	3.8 (0) (n=22)	3.8 (0) (n=42)	3.8 (0.17) (n=6)	3.8 (0) (n=112)	3.8 (0) (n=117)	0.061
Positive	91.8 (155.1) (n=151)	86.95 (207.7) (n=20)	109.5 (114.42) (n=8)	58.7 (108) (n=7)	95.4 (369.7) (n=7)	62.2 (150.05) (n=4)	93.8 (152.35) (n=81)	102.3 (250.92) (n=24)	0.92
p-value	< 0.001	< 0.001	< 0.001	< 0.001	< 0.001	0.067	< 0.001	< 0.001	
After First Dose									
Negative	8.77 (35.22) (n=230)	86.6 (60.6) (n=19)	94.95 (57.35) (n=14)	33.3 (75.75) (n=21)	13 (37.2) (n=41)	7.6 (4.45) (n=4)	4.05 (4.63) (n=108)	11.3 (22.71) (n=23)	< 0.001
Positive before vaccination	376 (1941) (n=127)	2945 (4882.4) (n=18)	3525 (8337.5) (n= 8)	2360 (3810) (n=7)	4160 (2690) (n=7)	191.5 (275.9) (n=4)	261 (325.75) (n=74)	2380 (2810) (n=9)	< 0.001
New cases	34.2 (-) (n=1)	–	–	–	34.2 (-) (n=1)	–	–	–	–
p-value	< 0.001	< 0.001	0.02	< 0.001	< 0.001	0.343	< 0.001	< 0.001	
After Second Dose									
Negative	163 (299.8) (n=305)	956 (833) (n=21)	1550 (952.5) (n=16)	1013 (2145) (n=22)	400 (2527) (n=41)	133.4 (170.3) (n=6)	114.5 (83.1) (n=94)	122 (181.2) (n=105)	< 0.001
Positive before vaccination	619.5 (2163.65) (n=132)	3155 (2707.5) (n=20)	2240 (7210) (n=7)	5640 (5736) (n=7)	6700 (2330) (n=7)	324.5 (401.2) (n=4)	249 (365.5) (n=65)	1385 (2872.5) (n=22)	< 0.001
New cases	400 (-) (n=3)	–	1960 (-) (n=1)	–	400 (-) (n=1)	–	180 (-) (n=1)	–	–
p-value	< 0.001	< 0.001	0.097	0.009	0.007	0.171	< 0.001	< 0.001	
Preboost Antibodies									
Negative	46 (107.2) (n=235)	195 (373) (n=11)	233.5 (1702.5) (n=6)	97.7 (146.75) (n=12)	176.5 (548) (n=40)	43.25 (59.22) (n=4)	25.2 (39.97) (n=68)	30.9 (51.3) (n=94)	< 0.001
Positive before vaccination	376 (745) (n=127)	886.5 (628.5) (n=12)	1695 (5360.75) (n=6)	184 (-) (n=3)	454 (2805.75) (n=6)	196 (-) (n=3)	303.5 (568.5) (n=63)	612 (1105.5) (n=21)	0.004
New cases	86.5 (1649.35) (n=44)	–	2430 (1665.5) (n=5)	–	360 (-) (n=1)	–	31.6 (298.8) (n=31)	654 (4693.7) (n=7)	0.012
p-value	< 0.001	0.013	0.103	0.295	0.303	0.057	< 0.001	< 0.001	
After Booster Dose									
Negative	1675 (3384) (n=152)	3940 (2097.5) (n=18)	1080 (2551.5) (n=5)	5700 (6012.5) (n=20)	467 (1038) (n=35)	3655 (1830) (n=4)	2570 (2147.5) (n=44)	575 (831.3) (n=26)	< 0.001
Positive before vaccination	2640 (2557.5) (n=100)	4005 (1770) (n=16)	2590 (0) (n=1)	11950(16372.5) (n=8)	1565 (9715.5) (n=6)	3380 (0) (n=2)	2240 (2070) (n=59)	1655 (2126) (n=8)	0.001
New cases	2760 (3310) (n=42)	5750 (-) (n=1)	2020 (-) (n=3)	–	1970 (11125) (n=5)	–	2795 (2495) (n=26)	2380 (1530) (n=7)	0.559
p-value	< 0.001	0.454	0.619	0.165	0.009	0.8	0.034	0.008	
Six months after Booster Dose									
Negative	368.5 (1606.5) (n=236)	326 (223.75) (n=18)	1035 (880.5) (n=10)	1764 (1540) (n=21)	1161 (2122.75) (n=34)	354 (1813) (n=5)	729.5 (1937.5) (n=58)	179 (228.25) (n=90)	< 0.001
Positive before vaccination	993 (1590) (n=157)	1160 (1475.75) (n=20)	797.5 (2215) (n=8)	2990 (4723.5) (n=8)	2500 (3642) (n=7)	1905 (2980.25) (n=4)	958.5 (1287) (n=86)	717 (953) (n= 24)	0.002
New cases	1605 (2160.25) (n=98)	3990 (1437.5) (n=4)	1740 (3745) (n=9)	–	825 (2736) (n=8)	2220 (-) (n=1)	1500 (2198) (n=49)	1790 (2341) (n=27)	0.18
p-value	< 0.001	0.001	0.404	0.032	0.454	0.293	0.047	< 0.001	
p-value	< 0.001	< 0.001	0.006	< 0.001	< 0.001	0.012	< 0.001	<0.001	

Data are presented as median and interquartile ranges. Mann–Whitney U, Kruskal–Wallis, and Friedman tests were used for comparison. A p-value less than 0.05 was considered significant.

Before vaccination there was no difference in the median (IQR) antibody titers between the vaccine groups, but there was a significant difference between patients with a negative versus a positive history of COVID-19 [3.8 (0) vs. 91.8 (155.1) AU/mL, p<0.001]. At around 21-28 days after the first dose, patients from the BNT162b2 + homologous booster and BNT162b2 + ChAdOx1-S schemes had the highest antibody titers for group 1 [86.6 (60.6) and 94.95 (57.35) AU/mL, respectively, p<0.001], whereas in group 2 the highest titer was by the Gam-COVID-Vac + ChAdOx1-S and BNT162b2 + ChAdOx1-S schemes [4160 (2690) and 3525 (8337.5) AU/mL, respectively, p<0.001]. There was only one new case during this time frame from the Gam-COVID-Vac + ChAdOx1-S scheme with 34.2 (-) AU/mL. Between 21-28 days after the second dose BNT162b2 + ChAdOx1-S and Gam-COVID-Vac + ChAdOx1-S continued to lead the highest quantities of median (IQR) antibodies for group 1 [1550 (952.5) and 1013 (2145) AU/mL, respectively, p<0.001], whereas in group 2 Gam-COVID-Vac + BNT162b2 and Gam-COVID-Vac + ChAdOx1-S had a higher median count when compared to the other vaccines [5640 (5736) and 6700 (2330) AU/mL, p<0.001]. There was a total of 3 new cases after the second dose with a median (range) of 400 (180-1960) AU/mL antibodies in the groups with BNT162b2 + ChAdOx1-S, Gam-COVID-Vac + ChAdOx1-S, and CoronaVac-ChAdOx1-S.

A decline in the number of antibodies can be seen four to six months after completion of the vaccination scheme by 71.7% in the first group and by 39.3% in the second. At this point both BNT162b2 + ChAdOx1-S and BNT162b2 + homologous booster maintained the highest antibody titers for the first group [233.5 (1702.5) and 195 (373) AU/mL, respec-tively, p<0.001], showing the same pattern for the second group [1695 (5360.75) and 886.5 (628.5) AU/mL, p=0.004]. New cases of infection during this period showed the highest median antibodies in the BNT162b2 + ChAdOx1-S and ChAdOx1-S + homologous booster vaccination schemes [2430 (1665.5) and 654 (4693.7) AU/mL, p=0.012].

Following the booster dose, the median of antibodies exhibited a remarkable 10.2-fold increase in group 1 compared to the levels observed after the second dose. For group 1, antibody titers were the highest in both Gam-COVID-Vac + BNT162b2 and BNT162b2 with homologous booster [5700 (6012.5) and 3940 (2097.5) AU/mL, respec-tively, p<0.001]. For group 2 there was a 4.2-fold increase in comparison to the parameters immediate after the completion of the scheme. The different schemes that showed the highest antibody titers for the second group were the same as the first group [11950 (16372.5) and 4005 (1770) AU/mL, respectively, p=0.001]. For the third group the BNT162b2 with homologous booster group showed the highest antibody titer, only being followed by the CoronaVac + ChAdOx1-S, but this showed a non-significant difference [5750 (-) and 2795 (2495) AU/mL, respectively, p=0.559].

**Figure 2:**
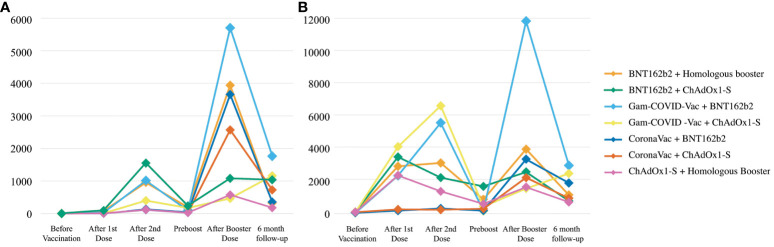
SARS-CoV-2 Spike 1–2 IgG antibodies over a six-month follow-up. **(A)** SARS-CoV-2 Spike 1–2IgG antibody levels (AU/mL) in subjects that were not infected with SARS-CoV-2 that were exposed to one of the six different types of vaccines. **(B)** SARS-CoV-2 Spike 1–2 IgG antibody levels (AU/mL) in subjects that were previously infected with SARS-CoV-2 before vaccination and that were exposed to one of the six different types of vaccines.

Six (+/- 1) months after the booster dose, an overall decrease of 78% was observed in the antibody titers from group 1. The groups Gam-COVID-Vac + BNT162b2 booster, Gam-COVID-Vac + ChAdOx1-S, and BNT162b2 + ChAdOx1-S booster showed the highest concentration of antibody titers [1764 (1540), 1161 (2122.75) and 1035 (880.5) AU/mL, respectively, p<0.001]. In group 2 there was a decrease of 62.3% of the antibodies from the immediate booster vaccine count. The highest antibody titers were seen in patients from the Gam-COVID-Vac + BNT162b2 booster, Gam-COVID-Vac + ChAdOx1-S booster, and CoronaVac + ChAdOx1-S booster groups [2990 (4723.5), 2500 (3642), and 1905 (2980.25) AU/mL, respectively, p=0.002]. Regarding group 3, of new cases through the follow up, the highest antibody counts were seen in the BNT162b2 with homologous booster, CoronaVac + BNT162b2 booster group, and ChAdOx1-S with homologous booster groups, but showing no significant differences between the groups [3990 (1437.5), 2220 (-), and 1790 (2341) AU/mL, respectively, p=0.18]. **Figure 2** provides a visual representation of the antibody response throughout the follow-up, categorized by the different evaluated vaccines.

### 3.3 Linear regression model for the antibody titers at 6-month follow-up after booster dose

We developed a linear regression model where the dependent variable was the antibody titers after 6 months from the booster dose. The independent variables included age, gender, vaccine combination, COVID-19 infection before vaccination, and COVID-19 infection after booster dose. COVID-19 infection before vaccination scheme (β=-1377.43, p<0.001) and COVID-19 infection after booster dose (β=1625.20, p<0.001) both led to higher antibody counts. Regarding the vaccination scheme, the primary Gam-COVID-Vac + BNT162b2 booster group is related to an increase in antibodies when compared to three doses of Pfizer (β=1223.91, p=0.004). **Table 4** shows the multiple regression model where the antibodies after 6 months from booster vaccination were the dependent variable.

**Table 4:** Multiple Linear Regression Model to determine prediction factors for SARS-CoV-2 Spike 1–2 IgG Antibodies 6 Months After the 3rd Dose.

Variable	β	Standard Error	t value	CI (Confidence Interval) 95%		p-value
				Lower	Upper	
Intercept	1012.48	476.22	2.126	77	1948	0.034
Age	0.951	8.83	0.108	-16	18	0.914
Male sex	67.22	138.39	0.49	-205	339	0.627
BNT162b2 + Homologous Booster						
BNT162b2 + ChAdOx1-S	474.62	381.93	1.24	-276	1225	0.215
Gam-COVID-Vac + BNT162b2	1223.91	417.20	2.93	404	2044	0.004
Gam-COVID-Vac + ChAdOx1-S	622.38	399.45	1.56	-163	1407	0.120
CoronaVac + BNT162b2	276.83	538.03	0.51	-780	1334	0.607
CoronaVac + ChAdOx1-S	26.79	262.50	0.10	-489	542	0.919
ChAdOx1-S + Homologous Booster	-385.90	353.36	-1.09	-1080	308	0.275
COVID-19 Infection before vaccination scheme	-1377.43	236.07	-5.83	-1841	-914	<0.001
COVID-19 Infection after booster dose	1625.20	219.85	7.39	1.193	2057	<0.001

Nagelkerke R^2^ = 0.141.

### 3.4 SARS-CoV-2 infection

Before vaccine applications a total of 151 (30.8%) patients contracted the COVID-19 infection, from which BNT162b2 + Homologous Booster [20 (47.6%)] and CoronaVac + ChAdOx1-S [81 (42%)] groups presented the majority of cases, in a proportional manner. The most frequent symptoms were headache [48 (31.8%)], myalgia [51 (33.8%)] and anosmia [49 (32.4%)]. From the 151 patients, 92 (60.9%) were treated in an ambulatory manner, 10 (6.6%) required hospitalization, and 1 (0.15%) was admitted to the Intensive Care Unit (ICU). A total of 7 (4.6%) requires supplemental oxygen, 4 requiring nasal cannula, 1 non-rebreather mask and 2 patients requiring intubation.

Between the application of the first dose and the second dose a total of 6 patients got infected, 2 from the CoronaVac + ChAdOx1-S group and the others from the ChAdOx1-S + Homologous Booster, BNT162b2 + Homologous Booster, BNT162b2 + ChAdOx1-S, and Gam-COVID-Vac + ChAdOx1-S groups. Half of the patients presented with fever and headache and 4 of the patients were treated ambulatorily while 1 patient required hospitalization without need for supplemental oxygen.

In the follow-up between the second and booster dose there were a total of 79 (16.1%) cases from which the majority were from CoronaVac + ChAdOx1-S [59 (30.6%)] and BNT162b2 + ChAdOx1-S [8 (29.6%)]. The most common symptoms were cough [50 (63.3%)], odynophagia [40 (50.6%)] and headache [37 (46,8%)]. The majority of patients were treated ambulatorily [70 (88.6%)], while a patient from the CoronaVac + ChAdOx1-S group and another from the ChAdOx1-S + Homologous Booster group required hospitalization, the latter of which required supplemental oxygen by nasal cannula.

In the follow-up after the booster dose there was a total of 73 (15%) cases from which the majority were from the CoronaVac + BNT162b2 [4 (40%)] and Coronavac + ChAdOx1-S [31 (16%)], just followed by Gam-COVID-Vac + ChAdOx1-S [8 (16%)]. The most common symptoms were odynophagya [37 (50.6%)], cough [29 (39.7%)] myalgias and tiredness [28 (38.3%)]. The presence of odynophagia, myalgia and tiredness were significantly prevalent in the group with CoronaVac + ChAdOx1-S compared to the other groups, p<0.05. All patients were treated ambulatorily, while just one patient from the ChAdOx-S + Homologous Booster required supplemental oxygenation through nasal cannula. **Table 5** shows in detail the presence of infection and symptoms through the follow up and the comparison between the different groups under study.

**Table 5:** SARS-CoV-2 Infection Before, In Between, and After Vaccination.

SARS-CoV-2 Infection	Total vaccines n= 491 (%)	BNT162b2 + Homologous Booster n= 42 (8.6%)	BNT162b2 + ChAdOx1-S n= 27 (5.5%)	Gam-COVID-Vac + BNT162b2 n= 29 (5.9%)	Gam-COVID-Vac + ChAdOx1-S n= 49 (10%)	CoronaVac + BNT162b2 n= 10 (2%)	CoronaVac + ChAdOx1-S n= 193 (39.3%)	ChAdOx1-S + Homologous Booster n= 141 (28.7%)	p-valor
Before Vaccination	151 (30,8%)	20 (47,6%)	8 (29,6%)	7 (24,1%)	7 (14,3%)	4 (40%)	81 (42%)	24 (17%)	<0,01
After First Dose	6 (1,2%)	1 (2,4%)	1 (3,7%)	0 (0%)	1 (2%)	0 (0%)	2 (1%)	1 (0,7%)	0,814
After Second Dose	79 (16,1%)	0 (0%)	8 (29,6%)	0 (0%)	1 (2%)	1 (10%)	59 (30,6%)	11 (7,8%)	<0,01
After Booster Dose	73 (15%)	5 (12%)	4 (15%)	2 (6,9%)	8 (16%)	4 (40%)	31 (16%)	19 (13%)	<0,01
SARS-CoV-2 Infection Before Vaccination									
Symptoms									
Fever	28 (18,5%)	2 (10%)	2 (25%)	0 (0%)	2 (28,6%)	1 (25%)	17 (20,9%)	4 (16,6%)	0,586
Feverish	27 (17,9%)	8 (40%)	2 (25%)	1 (14,3%)	1 (14,3%)	1 (25%)	13 (16%)	1 (4,2%)	0,054
Cough	41 (27,1%)	8 (40%)	2 (25%)	0 (0%)	1 (14,3%)	0 (0%)	23 (28,4%)	7 (29,2%)	0,762
Headache	48 (31,8%)	8 (40%)	2 (25%)	0 (0%)	1 (14,3%)	1 (25%)	29 (35,8%)	7 (29,2%)	0,861
Dyspnea	33 (21,8%)	3 (15%)	3 (37,5%)	1 (14,3%)	2 (28,6%)	0 (0%)	17 (20,9%)	7 (29,2%)	0,349
Conjuntivitis	5 (3,3%)	1 (5%)	0 (0%)	0 (0%)	1 (14,3%)	0 (0%)	3 (3,7%)	0 (0%)	0,447
Palpitations	16 (10,6%)	2 (10%)	0 (0%)	1 (14,3%)	1 (14,3%)	0 (0%)	9 (11,1%)	3 (12,5%)	0,674
Thoracic Pain	24 (15,9%)	5 (25%)	1 (12,5%)	0 (0%)	0 (0%)	0 (0%)	15 (18,5%)	3 (12,5%)	0,897
Odynophagia	41 (27,1%)	7 (25%)	2 (25%)	0 (0%)	2 (28,6%)	0 (0%)	24 (29,6%)	6 (25%)	0,828
Myalgias	51 (33,8%)	9 (45%)	2 (25%)	1 (14,3%)	2 (28,6%)	1 (25%)	26 (32,1%)	10 (41,7%)	0,939
Arthralgias	39 (25,8%)	9 (45%)	1 (12,5%)	1 (14,3%)	1 (14,3%)	1 (25%)	21 (25,9%)	5 (20,8%)	0,689
Anosmia	49 (32,4%)	8 (40%)	1 (12,5%)	1 (14,3%)	1 (14,3%)	1 (25%)	29 (35,8%)	8 (33,3%)	0,879
Tiredness	19 (12,6%)	6 (30%)	2 (25%)	1 (14,3%)	1 (14,3%)	1 (25%)	7 (8,6%)	1 (4,2%)	0,037
Diarrhea	16 (10,6%)	4 (20%)	0 (0%)	0 (0%)	1 (14,3%)	1 (25%)	8 (9,9%)	2 (8,3%)	0,606
Vomiting	9 (5,9%)	2 (10%)	0 (0%)	0 (0%)	1 (14,3%)	0 (0%)	4 (4,9%)	2 (8,3%)	0,653
Nausea	2 (1,3%)	0 (0%)	0 (0%)	0 (0%)	1 (14,3%)	0 (0%)	1 (1,2%)	0 (0%)	0,240
Treatment									
Ambulatory	92 (60,9%)	15 (75%)	3 (37,5%)	1 (14,3%)	2 (28,6%)	3 (75%)	53 (65,4%)	15 (62,5%)	0.012
Hospitalization	10 (6,6%)	1 (5%)	1 (12,5%)	1 (14,3%)	2 (28,6%)	0 (0%)	3 (3,7%)	2 (8,3%)	
Intensive Care Unit	1 (0,7%)	0 (0%)	1 (12,5%)	0 (0%)	0 (0%)	0 (0%)	0 (0%)	0 (0%)	
Need for supplementary oxygen									
Total	7 (4.6%)	1 (5%)	2 (25%)	0 (0%)	0 (0%)	0 (0%)	3 (3.7%)	1 (4.2%)	0,261
Nasal Cannula	4 (57.1%)	1 (100%)	1 (50%)	0 (0%)	0 (0%)	0 (0%)	1 (33.3%)	1 (100%)	1.000
Non-rebreather mask	1 (14,2%)	0 (0%)	0 (0%)	0 (0%)	0 (0%)	0 (0%)	1 (33.3%)	0 (0%)	
High flow equipment	0 (0%)	0 (0%)	0 (0%)	0 (0%)	0 (0%)	0 (0%)	0 (0%)	0 (0%)	
Orotracheal Intubation	2 (28.7%)	0 (0%)	1 (50%)	0 (0%)	0 (0%)	0 (0%)	1 (33.3%)	0 (0%)	
SARS-CoV-2 Infection After 1st Dose									
Symptoms									
Fever	3 (50%)	0 (0%)	1 (100%)	0 (0%)	1 (100%)	0 (0%)	1 (50%)	0 (0%)	1,000
Feverish	2 (33,3%)	0 (0%)	0 (0%)	0 (0%)	0 (0%)	0 (0%)	2 (100%)	0(0%)	0,401
Cough	2 (33,3%)	0 (0%)	0 (0%)	0 (0%)	0 (0%)	0 (0%)	2 (100%)	0(0%)	0,401
Headache	3 (50%)	0 (0%)	1 (100%)	0 (0%)	0 (0%)	0 (0%)	1 (50%)	1 (100%)	1,000
Dyspnea	1 (16,6%)	0 (0%)	1 (100%)	0 (0%)	0 (0%)	0 (0%)	0 (0%)	0 (0%)	0,602
Conjuntivitis	0 (0%)	0 (0%)	0 (0%)	0 (0%)	0 (0%)	0 (0%)	0 (0%)	0 (0%)	-
Palpitations	1 (16,6%)	0 (0%)	0 (0%)	0 (0%)	0 (0%)	0 (0%)	1 (50%)	0 (0%)	1,000
Thoracic Pain	0 (0%)	0 (0%)	0 (0%)	0 (0%)	0 (0%)	0 (0%)	0 (0%)	0 (0%)	-
Odynophagia	2 (33,3%)	0 (0%)	1 (100%)	0 (0%)	0 (0%)	0 (0%)	1 (50%)	0 (0%)	1,000
Myalgias	0 (0%)	0 (0%)	0 (0%)	0 (0%)	0 (0%)	0 (0%)	0 (0%)	0 (0%)	-
Arthralgias	2 (33,3%)	0 (0%)	0 (0%)	0 (0%)	0 (0%)	0 (0%)	1 (50%)	1 (100%)	1,000
Anosmia	1 (16,6%)	0 (0%)	0 (0%)	0 (0%)	0 (0%)	0 (0%)	1 (50%)	0 (0%)	1,000
Tiredness	2 (33,3%)	0 (0%)	0 (0%)	0 (0%)	1 (100%)	0 (0%)	1 (50%)	0 (0%)	1,000
Diarrhea	1 (16,6%)	0 (0%)	0 (0%)	0 (0%)	0 (0%)	0 (0%)	1 (50%)	0 (0%)	1,000
Vomiting	1 (16,6%)	0 (0%)	0 (0%)	0 (0%)	0 (0%)	0 (0%)	1 (50%)	0 (0%)	1,000
Nausea	1 (16,6%)	0 (0%)	0 (0%)	0 (0%)	0 (0%)	0 (0%)	0 (0%)	1 (100%)	0,602
Treatment									
Ambulatory	4 (66,6%)	0 (0%)	1 (100%)	0 (0%)	1 (100%)	0 (0%)	1 (50%)	1 (100%)	1,000
Hospitalization	1 (16,6%)	0 (0%)	0 (0%)	0 (0%)	0 (0%)	0 (0%)	1 (50%)	0 (0%)	1,000
Intensive Care Unit	0 (0%)	0 (0%)	0 (0%)	0 (0%)	0 (0%)	0 (0%)	0 (0%)	0 (0%)	-
Need for supplementary oxygen									
Total	0 (0%)	0 (0%)	0 (0%)	0 (0%)	0 (0%)	0 (0%)	0 (0%)	0 (0%)	-
Nasal Cannula	0 (0%)	0 (0%)	0 (0%)	0 (0%)	0 (0%)	0 (0%)	0 (0%)	0 (0%)	-
Non-rebreather mask	0 (0%)	0 (0%)	0 (0%)	0 (0%)	0 (0%)	0 (0%)	0 (0%)	0 (0%)	-
High flow equipment	0 (0%)	0 (0%)	0 (0%)	0 (0%)	0 (0%)	0 (0%)	0 (0%)	0 (0%)	-
Orotracheal Intubation	0 (0%)	0 (0%)	0 (0%)	0 (0%)	0 (0%)	0 (0%)	0 (0%)	0 (0%)	-
SARS-CoV-2 Infection After 2nd Dose									
Symptoms									
Fever	18 (22,8%)	0 (0%)	2 (25%)	0 (0%)	0 (0%)	1 (100%)	14 (23,7%)	1 (9,1%)	0,270
Feverish	12 (15,2%)	0 (0%)	1 (12,5%)	0 (0%)	0 (0%)	0 (0%)	10 (16,9%)	1 (9,1%)	1,000
Cough	50 (63,3%)	0 (0%)	4 (50%)	0 (0%)	0 (0%)	0 (0%)	41 (69,5%)	5 (45,5%)	0,150
Headache	37 (46,8%)	0 (0%)	4 (50%)	0 (0%)	0 (0%)	1 (100%)	30 (50,8%)	2 (18,2%)	0,143
Dyspnea	3 (3,8%)	0 (0%)	0 (0%)	0 (0%)	0 (0%)	0 (0%)	2 (3,4%)	1 (9,1%)	0,589
Conjuntivitis	3 (3,8%)	0 (0%)	0 (0%)	0 (0%)	0 (0%)	0 (0%)	2 (3,4%)	1 (9,1%)	0,589
Palpitations	3 (3,8%)	0 (0%)	2 (25%)	0 (0%)	0 (0%)	0 (0%)	1 (1,7%)	0 (0%)	0,049
Thoracic Pain	7 (8,9%)	0 (0%)	0 (0%)	0 (0%)	0 (0%)	0 (0%)	6 (10,2%)	1 (9,1%)	1,000
Odynophagia	40 (50,6%)	0 (0%)	5 (62,5%)	0 (0%)	0 (0%)	0 (0%)	30 (50,8%)	5 (45,5%)	0,862
Myalgias	33 (41,8%)	0 (0%)	2 (25%)	0 (0%)	0 (0%)	1 (100%)	28 (47,5%)	2 (18,2%)	0,108
Arthralgias	23 (29,1%)	0 (0%)	2 (25%)	0 (0%)	0 (0%)	0 (0%)	18 (30,5%)	3 (27,3%)	1,000
Anosmia	15 (19%)	0 (0%)	1 (12,5%)	0 (0%)	0 (0%)	0 (0%)	12 (20,3%)	2 (18,2%)	1,000
Tiredness	19 (24,1%)	0 (0%)	3 (37,5%)	0 (0%)	0 (0%)	0 (0%)	13 (22%)	3 (27,3%)	0,696
Diarrhea	5 (6,3%)	0 (0%)	1 (12,5%)	0 (0%)	0 (0%)	0 (0%)	3 (5,1%)	1 (9,1%)	0,696
Vomiting	1 (1,3%)	0 (0%)	0 (0%)	0 (0%)	0 (0%)	0 (0%)	1 (1,7%)	0 (0%)	1,000
Nausea	0 (0%)	0 (0%)	0 (0%)	0 (0%)	0 (0%)	0 (0%)	0 (0%)	0 (0%)	-
Treatment									
Ambulatory	70 (88,6%)	0 (0%)	8 (100%)	0 (0%)	0 (0%)	1 (100%)	58 (98,3%)	10 (90,9%)	0,441
Hospitalization	2 (2,5%)	0 (0%)	0 (0%)	0 (0%)	0 (0%)	0 (0%)	1 (1,7%)	1 (9,1%)	
Intensive Care Unit	0 (0%)	0 (0%)	0 (0%)	0 (0%)	0 (0%)	0 (0%)	0 (0%)	0 (0%)	
Need for supplementary oxygen									
Total	1 (1,3%)	0 (0%)	0 (0%)	0 (0%)	0 (0%)	0 (0%)	0 (0%)	1 (9,1%)	0,509
Nasal Cannula	1 (1,3%)	0 (0%)	0 (0%)	0 (0%)	0 (0%)	0 (0%)	0 (0%)	1 (9,1%)	0,249
Non-rebreather mask	0 (0%)	0 (0%)	0 (0%)	0 (0%)	0 (0%)	0 (0%)	0 (0%)	0 (0%)	
High flow equipment	0 (0%)	0 (0%)	0 (0%)	0 (0%)	0 (0%)	0 (0%)	0 (0%)	0 (0%)	
Orotracheal Intubation	0 (0%)	0 (0%)	0 (0%)	0 (0%)	0 (0%)	0 (0%)	0 (0%)	0 (0%)	
SARS-CoV-2 Infection After 3rd Dose									
Symptoms									
Fever	19 (26%)	0 (0%)	3 (4,1%)	0 (0%)	0 (0%)	1 (1,4%)	12 (16,4%)	3 (4,1%)	0,066
Feverish	11 (15%)	0 (0%)	1 (1,4%)	0 (0%)	0 (0%)	3 (4,1%)	6 (8,2%)	1 (1,4%)	0,043
Cough	29 (39,7%)	1 (1,4%)	4 (5,5%)	0 (0%)	1 (7,1%)	1 (25%)	15 (20,5%)	7 (9,6%)	0,180
Headache	27 (36,9%)	2 (2,7%)	2 (2,7%)	0 (0%)	4 (5,5)	4 (5,5%)	12 (16,4%)	3 (4,1%)	0,053
Dyspnea	4 (5,4%)	1 (1,4%)	0 (0%)	0 (0%)	0 (0%)	0 (0%)	2 (2,7%)	1 (1,4%)	0,791
Conjuntivitis	5 (6,8%)	0 (0%)	0 (0%)	0 (0%)	0 (0%)	0 (0%)	3 (4,1%)	2 (2,7%)	1,000
Palpitations	4 (5,4%)	1 (1,4%)	0 (0%)	0 (0%)	0 (0%)	1 (1,4%)	0 (0%)	2 (2,7%)	0,096
Thoracic Pain	3 (4,1%)	0 (0%)	0 (0%)	0 (0%)	0 (0%)	0 (0%)	2 (2,7%)	1 (1,4%)	1,000
Odynophagia	37 (50,6%)	2 (2,7%)	3 (4,2%)	1 (1,4%)	0 (0%)	4 (5,5%)	20 (27,4%)	7 (9,6%)	0,005
Myalgias	28 (38,3%)	0 (0%)	4 (5,5%)	2 (2,7%)	4 (5,5%)	3 (4,1%)	12 (16,4%)	3 (4,1%)	0,008
Arthralgias	19 (26%)	0 (0%)	2 (2,7%)	2 (2,7%)	1 (1,4%)	2 (2,7%)	9 (12,3%)	3 (4,1%)	0,106
Anosmia	9 (12,3%)	0 (0%)	0 (0%)	0 (0%)	0 (0%)	1 (1,4%)	6 (8,2%)	2 (2,7%)	0,665
Tiredness	28 (38,3%)	1 (1,4%)	0 (0%)	2 (2,7%)	7 (9,6%)	1 (1,4%)	10 (13,7%)	7 (9,6%)	0,012
Diarrhea	1 (1,3%)	0 (0%)	1 (1,4%)	0 (0%)	0 (0%)	0 (0%)	0 (0%)	0 (0%)	0,216
Vomiting	2 (2,7%)	0 (0%)	0 (0%)	0 (0%)	0 (0%)	1 (1,4%)	1 (1,4%)	0 (0%)	0,290
Nausea	0 (0%)	0 (0%)	0 (0%)	0 (0%)	0 (0%)	0 (0%)	0 (0%)	0 (0%)	–
Treatment									
Ambulatory	73 (100%)	5 (100%)	5 (100%)	2 (100%)	8 (100%)	4 (100%)	31 (100%)	18 (100%)	-
Hospitalization	0 (0%)	0 (0%)	0 (0%)	0 (0%)	0 (0%)	0 (0%)	0 (0%)	0 (0%)	
Intensive Care Unit	0 (0%)	0 (0%)	0 (0%)	0 (0%)	0 (0%)	0 (0%)	0 (0%)	0 (0%)	
Need for supplementary oxygen									
Total	1 (1,4%)	0 (0%)	0 (0%)	0 (0%)	0 (0%)	0 (0%)	0 (0%)	1 (5.55%)	0,574
Nasal Cannula	1 (100%)	0 (0%)	0 (0%)	0 (0%)	0 (0%)	0 (0%)	0 (0%)	1 (100%)	-
Non-rebreather mask	0 (0%)	0 (0%)	0 (0%)	0 (0%)	0 (0%)	0 (0%)	0 (0%)	0 (0%)	
High flow equipment	0 (0%)	0 (0%)	0 (0%)	0 (0%)	0 (0%)	0 (0%)	0 (0%)	0 (0%)	
Orotracheal Intubation	0 (0%)	0 (0%)	0 (0%)	0 (0%)	0 (0%)	0 (0%)	0 (0%)	0 (0%)	

Data are presented as frequencies and percentages. Fisher’s Exact test with Monte Carlo approximation was used for comparison. A p-value <0.05 was considered statistically significant.

### 3.5 Survival analysis and Cox proportional hazard model

**Figure 3:**
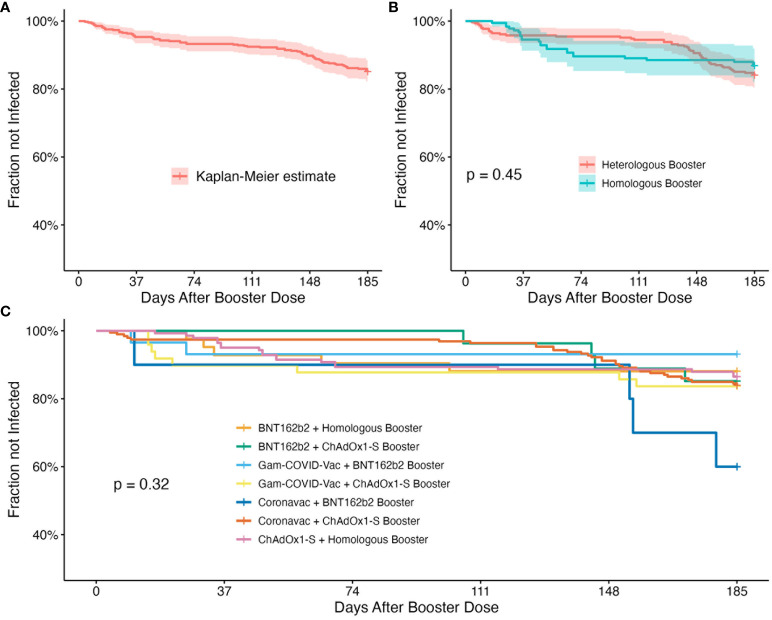
Survival analysis curves over 185 days follow-up after the application of the booster dose. **(A)** Kaplan-Meier estimate for the whole population. **(B)** Kaplan-Meier of the different vaccine schemes grouped into heterologous and homologous boosters. Heterologous booster group includes BNT162b2 + ChAdOx1-S booster, Gam-COVID-Vac + ChAdOx1-S booster, Gam-COVID-Vac + BNT162b2 booster, CoronaVac + BNT162b2 booster, CoronaVac + ChAdOx1-S booster, while the homologous booster group included the BNT162b2 and ChAdOx1-S vaccines with an homologous booster; statistical significance was calculated by Log-Rank Test. **(C)** Kaplan-Meier by the different vaccine groups, with statistical significance calculated by Log-Rank Test.

The adjusted Cox proportional hazard model involved 491 patients, with 73 developing a COVID-19 infection post-booster dose administration. Notably, a history of prior COVID-19 infection before vaccination was associated with a decreased risk of infection (HR= -0.90, p=0.004). However, factors such as age, sex, arterial hypertension, type 2 diabetes, and the type of vaccine applied were not significant in the model. The survival curves indicated no discernible difference between the various vaccine groups concerning the fraction of infected patients. This observation held true even when vaccines were grouped by heterologous versus homologous categories. The complete results are shown in **Table 6** and survival curves are shown in **Figure 3**.

**Table 6:** Adjusted Cox Proportional Hazard Model for Risk Factors for Mortality.

Variables	B	Standard Error	p-value	HR	95% CI	
					Lower	Upper
Age	-0.016	*0.015*	0.266	-0.02	-0.05	0.01
Male	0.278	*0.239*	0.245	0.28	-0.19	0.75
BNT162b2 with homologous booster-			-			
BNT162b2 + ChAdOx1-S booster	-0.185	*0.685*	0.788	-0.18	-1.5	1.2
Gam-COVID-Vac + BNT162b2 booster	-0.401	*0.898*	0.655	-0.40	-2.2	1.4
Gam-COVID-Vac + ChAdOx1-S booster	0.637	*0.711*	0.370	0.64	-0.76	2.0
CoronaVac + BNT162b2 booster	1.291	*0.684*	0.059	1.3	-0.05	2.6
CoronaVac + ChAdOx1-S booster	0.312	*0.493*	0.526	0.31	-0.65	1.3
ChAdOx1-S with homologous booster	0.362	*0.641*	0.572	0.36	-0.89	1.6
Hypertension	-0.205	*0.275*	0.455	-0.21	-0.74	0.33
Type 2 Diabetes Mellitus	-0.105	*0.335*	0.753	-0.11	-0.76	0.55
COVID-19 infection before vaccination scheme	-0.899	*0.315*	0.004	-0.90	-1.5	-0.28

HR, hazard ratio; CI, Confidence Interval. Reference from Male is Female, reference from the vaccine groups is the BNT162b2 plus homologous booster. Dependent Variable is COVID-19 Infection after the booster dose.

## 4 Discussion

In this study, we evaluated and compared the humoral response and protection elicited by SARS-CoV-2 vaccines following either a heterologous or homologous booster dose administered after a primary homologous scheme. The study encompassed a total of seven distinct groups, comprising 491 patients. Our analysis extended to the assessment of Adverse Events Following Immunization (AEFI), infection symptoms, and predictors of infections across these seven groups, within a follow-up period of 185 days after the booster dose.

### 4.1 Reactogenicity after the booster dose

The incidence of Adverse Events Following Immunization (AEFI) did not exhibit a significant increase as patients moved from the second dose of the primary scheme to the booster dose. Notably, vaccine combinations with CoronaVac as the primary component showed a higher prevalence of AEFI, while patients with a primary scheme of Gam-COVID-Vac + ChAdOx1-S booster had the lowest rate of adverse events. The severity of reactions was particularly pronounced in the CoronaVac + BNT162b2 and CoronaVac + ChAdOx1-S groups, especially with ChAdOx1-S as the booster. Subsequent to booster administration, there was a general rise in the number of moderate reactions compared to the first and second doses, primarily driven by the CoronaVac + ChAdOx1-S combination.

Previous studies have yielded conflicting results regarding AEFIs. In a study in Thailand involving 875 patients divided into nearly equivalent groups, one with primary and booster doses of CoronaVac and the other with CoronaVac + ChAdOx1-S, showed that patients with the latter combination had significantly lower AEFIs (18.09% vs. 6.43%, p=0.001). Conversely, a study in Brazil in which patients with a primary CoronaVac were divided into groups by either heterologous or homologous booster, indicated a higher prevalence of AEFIs in the heterologous groups compared to the homologous ones, concluding also that the primary CoronaVac + ChAdOx1-S booster group presented greater severity across different symptoms compared to the other vaccine combinations (25, 26). The inconsistency between these two studies could be attributed to differences in population demographics, vaccination protocols, and overall vaccine effectiveness in each respective region and could be the reason for our data to align better with the Brazilian study results. These inconsistencies were assessed in a meta-analysis of 5870 patients, which concluded that, as long as the booster dose was comprised of either an mRNA vaccine or a virus vector vaccine, the incidence of adverse events in the population would be higher (27).

Patients in the groups with Gam-COVID-Vac as the primary vaccine and either BNT162b2 or ChAdOx1-S as the booster experienced only very mild reactions. To the best of our knowledge, no prior studies have explored the reactogenicity of Gam-COVID-Vac primary vaccine plus a heterologous boost. However, earlier research on Gam-COVID-Vac in primary heterologous schemes (mix and matched vaccines) revealed that patients who received this vaccine plus a vaccine from either ChAdOx1-S or mRNA-1273 had only local reactions, not systemic ones (28). A study protocol in Iran has been developed on this specific topic, but results have not been published (29).

### 4.2 Humoral response to the booster dose

In relation to the humoral response following the administration of the booster vaccine, individuals with a history of infection either preceding the initiation of any vaccination or during the follow-up, displayed a markedly elevated overall antibody titer production. This finding aligns with multiple prior studies that have documented similar outcomes in the monitoring of vaccination regimens, suggesting a potentiation of the immune response generated initially after COVID-19 infection (22, 30).

The median antibody production, spanning from the completion of the vaccination regimen to the booster dose, manifested an approximate 7.4-fold increase, indicating a cumulative rise in antibody titers with each subsequent immunization. However, six months after the booster dose, a noticeable decline in antibody titers was observed, with the most pronounced reduction observed in the group without prior infection compared to the previously infected group, which resembles the waning seen in the preboost measurement. In a study involving 405 healthcare workers who received a homologous booster dose of BNT162b2, the post-booster response exhibited a higher magnitude, primarily determined by the quantity of antibodies post-completion of the vaccination scheme. The study claimed that these patients experienced a more gradual waning of antibodies compared to the decline witnessed after the second dose, a trend not observed in our population (31). Similarly, another study involving 113 healthcare workers, stratified based on primary vaccination (BNT162b2, ChAdOx1-S, or mRNA-1273) and subsequent homologous or heterologous boosters, found that a homologous mRNA-1273 booster exhibited a slower decline in antibody titers, followed closely by homologous BNT162b2, with the ChAdOx1-S + BNT162b2 booster at the end (32). Consistent evidence across multiple studies corroborates the phenomenon of antibodies experiencing a steady waning process in the four to six months following the administration of the booster dose (33, 34).

Notably, the primary vaccination with BNT162b2 showcased the highest median antibody levels before booster dose, regardless of individuals’ prior infection status. Following the booster dose, a remarkable 10.2-fold increase was observed in the naive group, and a 4.2-fold increase in the previously infected group. The Gam-COVID-Vac + BNT162b2 group exhibited the highest median antibody levels, closely followed by BNT162b2 with a homologous booster, for patients with and without a history of infection. Despite an overall decline of approximately 70% in antibody titers six months post-booster, the Gam-COVID-Vac + BNT162b2 booster and Gam-COVID-Vac + ChAdOx1-S groups consistently maintained higher antibody levels than other vaccine combinations, by this showing a slower waning. Previous research has consistently highlighted the superior immunogenicity and efficacy rates of mRNA-based COVID-19 vaccines, such as BNT162b2, during the primary vaccination scheme (35, 36). This sustained higher immunogenicity of BNT162b2 remains even after the booster dose, and has been corroborated in a study involving 352 participants with either CoronaVac or ChAdOx-1 exposed to a booster dose from various vaccines, where a combination with BNT162b2, at either full or half dose, elicited a consistently superior response (37). Furthermore, a network meta-analysis of nine randomized controlled trials concluded that patients receiving a booster of BNT162b2 generate a greater response and higher levels of neutralizing antibodies compared to homologous boosters of non-mRNA vaccines (38). However, intriguingly, the vaccine group displaying higher antibodies six months post-booster were those who had Gam-COVID-Vac as the primary booster. There is a lack of prior studies elucidating antibody titer behavior after a booster dose for patients who had this specific vaccine. The unique mechanism of action of Gam-COVID-Vac, utilizing a replication-deficient adenovirus as a vector with the distinctive use of two adenovirus types (Ad26 and Ad5), differs from other virus-vector vaccines. A booster with either another vector-viral vaccine, such as the replication-deficient chimpanzee adenoviral vector ChAdOx1, or an mRNA vaccine like BNT162b2, could generate a synergistic effect, potentially enhancing immune system activation and providing a more enduring defense against COVID-19 (39–41).

The regression analysis conducted revealed that key variables predicting antibody levels six months post-booster were a positive history of infection before any vaccination scheme and a COVID-19 infection after the booster dose. Notably, the primary Gam-COVID-Vac vaccination scheme plus BNT162b2 booster emerged as a significant predictor, indicating higher antibody titers among vaccination groups. Contrary to some prior studies, age and sex were found to be unrelated to the immunogenicity generated by the vaccine in our analysis. This contrasts with a relevant study involving 514 Israeli healthcare workers after a single BNT162b2 dose, where no significant relation was found between either sex or ethnicity and the humoral response, but highlighted age as a crucial factor in COVID-19 vaccine immunogenicity. Older individuals exhibited a diminished immune response compared to their younger counterparts, a trend first observed in animal models (42, 43).

### 4.3 Effectiveness against SARS-CoV-2 infection after the booster dose

There was a small decrease in the number of infected patients from the second dose to the booster dose, going from an infection rate of 16.1% to 15%. The most common symptom presented by infected patients after the booster dose was odynophagia followed by cough and myalgia/tiredness, while in contrast cough was the main complaint during infection following the second dose, followed by odynophagia, headache and myalgia. Interestingly, a Chinese study on the clinical profiles of patients with breakthrough Omicron-variant infection found a similar pattern, with tiredness being more common in after-booster infection and cough being more common in patients infected after primary immunization (ie. two doses). Similar to our findings, odynophagia exhibited higher prevalence in booster patients, accompanied by a reduction in cough. However, contrary to our results, fever emerged as the most frequent manifestation for both groups in their study (44). These variations in clinical presentation might be attributed to diverse factors, such as the viral variants affecting our population or the demographic characteristics unique to each population. Notably, our study had a median (IQR) age of 57 (23), while only 10% of patients in the Chinese sample were 46 years or older.

Regarding the likelihood of infection by means of survival analysis, data revealed that variables such as age, sex, arterial hypertension, and type 2 diabetes did not demonstrate significant differences. Moreover, specific vaccine combinations did not exhibit superiority, as survival curves showed no distinctions, even when categorizing vaccination as either heterologous or homologous. The sole predictor of infection was a prior COVID-19 infection before the primary vaccination scheme, which correlated with a reduced likelihood of contracting the disease. Despite the group receiving the primary Gam-COVID-Vac + BNT162b2 booster maintaining the highest antibody titer response after six months and having the lowest infection rate, this did not translate into a lower infection rate prediction. In contrast to our findings, a meta-analysis of 23 studies involving over a million patients indicated higher vaccine effectiveness against SARS-CoV-2 in those with a heterologous combination (96.1%) compared to homologous combinations (84.0%). Interestingly, the same study found similar protection against hospital admission for both heterologous and homologous combinations (97.4% vs. 93.2%), an outcome we weren’t able to examine since none of our patients required hospitalization (45). Another study, utilizing data sources from four European countries and a 1-to-1 matched cohort study on BNT162b2, mRNA-1273 as boosters for homologous and heterologous schemes (BNT162b2, mRNA-1273, ChAdOx1-S), demonstrated high vaccine effectiveness against infection in both homologous boosters (42-88%) and heterologous boosters (70-86%) (46). Our study aligns with the notion that vaccines provide protection against infection and severity but differs in suggesting that one vaccine group may be superior to others. The divergent conclusions among various studies highlight the heterogeneity resulting from the creation and combination of different vaccines, underscoring the need for exploration of less-researched vaccines, such as Gam-COVID-Vac.

This study represents a real-world investigation into booster vaccines, focusing on a broader range of vaccination combinations, including less commonly studied vaccine combinations. We examined these vaccines in a population with persistent challenges related to vaccination—specifically, Mexico and Argentina. Our findings reveal that, regardless of vaccine category as homologous or heterologous, there was no significant change in infectivity rate. Moreover, after a six-month follow-up, no vaccination group demonstrated superior effectiveness compared to the others. This real-world study had inherent limitations, primarily the variability in the number of patients within each vaccine combination group, making it challenging to draw definitive conclusions for combinations with smaller sample sizes, such as CoronaVac + BNT162b2. Additionally, our study would benefit from a more diverse range of booster vaccines beyond Pfizer and AstraZeneca, providing a more comprehensive understanding of vaccine dynamics in different combinations.

## 5 Conclusions

Both homologous and heterologous COVID-19 booster doses exhibit high effectiveness, immunogenicity, and acceptable safety profiles. A higher incidence of Adverse Events Following Immunization was observed in the primary CoronaVac + ChAdOx1-S booster group, while patients with a primary BNT162b2 scheme, whether with a homologous or heterologous booster, displayed a greater production of antibodies. A sustained and higher level of antibodies was maintained in patients with a primary Gam-COVID-Vac vaccine, regardless of whether the booster was BNT162b2 or ChAdOx1-S. These insights into the safety and efficacy profiles of different vaccine combinations contribute valuable information to the ongoing discourse on optimal vaccination strategies.

## Data availability statement

The raw data supporting the conclusions of this article will be made available by the authors, without undue reservation.

## Ethics statement

The studies involving humans were approved by COMITÉ DE ETICA EN INVESTIGACION DE LA ESCUELA DE MEDICINA DE LA UNIVERSIDAD DE MONTERREY/COMITÉ DE ETICA EN INVESTIGACIÓN DE HOSPITAL INTERZONAL GENERAL DE AGUDOS SAN FELIPE. The studies were conducted in accordance with the local legislation and institutional requirements. The participants provided their written informed consent to participate in this study.

## Author contributions

AG: Data curation, Formal analysis, Investigation, Software, Validation, Writing – original draft, Writing – review & editing. DR: Data curation, Investigation, Writing – original draft, Writing – review & editing. AR: Data curation, Investigation, Writing – original draft, Writing – review & editing. IF: Formal analysis, Investigation, Software, Validation, Writing – original draft, Writing – review & editing. AC: Formal analysis, Investigation, Software, Validation, Writing – original draft, Writing – review & editing. DM: Investigation, Writing – original draft, Writing – review & editing. IB: Investigation, Project administration, Writing – original draft, Writing – review & editing. MS: Conceptualization, Investigation, Project administration, Resources, Writing – original draft, Writing – review & editing. CA: Investigation, Resources, Writing – original draft, Writing – review & editing. GP: Investigation, Writing – original draft, Writing – review & editing. MT: Conceptualization, Investigation, Methodology, Resources, Supervision, Visualization, Writing – original draft, Writing – review & editing. EA: Investigation, Methodology, Writing – original draft, Writing – review & editing. CP: Investigation, Methodology, Writing – original draft, Writing – review & editing. MR: Conceptualization, Investigation, Methodology, Resources, Supervision, Visualization, Writing – original draft, Writing – review & editing. MR: Conceptualization, Formal analysis, Investigation, Methodology, Project administration, Software, Supervision, Validation, Visualization, Writing – original draft, Writing – review & editing.

## References

[1] Balboa-Castillo T Andrade-Mayorga O Marzuca-Nassr GN Morales Illanes G Ortiz M Schiferlli I. Pre-existing conditions in Latin America and factors associated with adverse outcomes of COVID-19: A review. Medwave. (2021) 21:e8180. doi: 10.5867/medwave.2021.04.8180 34037583

[2] Rodriguez-Morales AJ Bonilla-Aldana DK. Epidemiology of COVID-19 in Latin America. In: Pandemic Outbreaks in the 21st Century. Netherlands: Elsevier (2021). p. 11–24. Available at: https://linkinghub.elsevier.com/retrieve/pii/B9780323856621000124.

[3] Fernandez-Guzman D Chavez-Cruzado E Diaz-Velez C Galvez-Olortegui T Vergara-de La Rosa E Rodríguez-Morales AJ. Advances and Challenges in COVID-19 Vaccination in Latin American: A public health perspective. Infect. (2022) 26(4):441–9. doi: 10.22354/24223794.1094

[4] Liang LL Kuo HS Ho HJ Wu CY. COVID-19 vaccinations are associated with reduced fatality rates: Evidence from cross-county quasi-experiments. J Glob Health. (2021) 11:05019. doi: 10.7189/jogh.11.05019 34326999 PMC8285768

[5] Damijan JP Damijan S Kostevc Č. Vaccination is reasonably effective in limiting the spread of COVID-19 infections, hospitalizations and deaths with COVID-19. Vaccines. (2022) 10:678. doi: 10.3390/vaccines10050678 35632434 PMC9143604

[6] World Health Organization. Cumulative confirmed COVID-19 deaths by world region (2024). Available online at: https://ourworldindata.org/grapher/cumulative-covid-cases-region?tab=table.

[7] World Health Organization. Total COVID-19 vaccine doses administered per 100 people (2024). Available online at: https://ourworldindata.org/grapher/covid-vaccination-doses-per-capita?tab=map&time=2023-12-31&country=~BRA.

[8] Rosenblum HG Wallace M Godfrey M Roper LE Hall E Fleming-Dutra KE. Interim recommendations from the advisory committee on immunization practices for the use of bivalent booster doses of COVID-19 vaccines — United states, october 2022. MMWR Morb Mortal Wkly Rep. (2022) 71:1436–41. doi: 10.15585/mmwr.mm7145a2 PMC9707353 36355612

[9] World Health Organization. WHO SAGE roadmap for prioritizing uses of COVID-19 vaccines: an approach to optimize the global impact of COVID-19 vaccines, based on public health goals, global and national equity, and vaccine access and coverage scenarios. Geneva: World Health Organisation (2022). Available at: https://iris.who.int/handle/10665/351138.

[10] Atmar RL Lyke KE Deming ME Jackson LA Branche AR El Sahly HM. Homologous and heterologous covid-19 booster vaccinations. N Engl J Med. (2022) 386:1046–57. doi: 10.1056/NEJMoa2116414 PMC8820244 35081293

[11] Suah JL Tng BH Tok PSK Husin M Thevananthan T Peariasamy KM. Real-world effectiveness of homologous and heterologous BNT162b2, CoronaVac, and AZD1222 booster vaccination against Delta and Omicron SARS-CoV-2 infection. Emerg Microbes Infect. (2022) 11:1343–5. doi: 10.1080/22221751.2022.2072773 PMC9132393 35499301

[12] Kyaw MH Spinardi J Zhang L Oh HML Srivastava A. Evidence synthesis and pooled analysis of vaccine effectiveness for COVID-19 mRNA vaccine BNT162b2 as a heterologous booster after inactivated SARS-CoV-2 virus vaccines. Hum Vaccines Immunother. (2023) 19:2165856. doi: 10.1080/21645515.2023.2165856 PMC9980688 36727201

[13] Zuo F Abolhassani H Du L Piralla A Bertoglio F De Campos-Mata L. Heterologous immunization with inactivated vaccine followed by mRNA-booster elicits strong immunity against SARS-CoV-2 Omicron variant. Nat Commun. (2022) 13:2670. doi: 10.1038/s41467-022-30340-5 35562366 PMC9106736

[14] Wanlapakorn N Suntronwong N Kanokudom S Assawakosri S Nilyanimit P Yorsaeng R. Immunogenicity of the BNT162b2 COVID-19 vaccine as a third dose (booster) following two doses of different primary series regimens in Thailand. Pathog Global Health. (2022) 116:395–7. doi: 10.1080/20477724.2022.2108646 PMC9518238 35920191

[15] Cerqueira-Silva T Katikireddi SV De Araujo Oliveira V Flores-Ortiz R Júnior JB Paixão ES. Vaccine effectiveness of heterologous CoronaVac plus BNT162b2 in Brazil. Nat Med. (2022) 28:838–43. doi: 10.1038/s41591-022-01701-w PMC9018414 35140406

[16] Marra AR Miraglia JL Malheiros DT Guozhang Y Teich VD Da Silva Victor E. Effectiveness of heterologous coronavirus disease 2019 (COVID-19) vaccine booster dosing in Brazilian healthcare workers, 2021. Clin Infect Dis. (2023) 76:e360–6. doi: 10.1093/cid/ciac430 PMC9213833 35639918

[17] Romero-Ibarguengoitia ME Rivera-Salinas D Hernández-Ruíz YG Armendariz-Vázquez AG González-Cantú A Barco-Flores IA. Effect of heterologous vaccination regimen with ad5-nCoV canSinoBio and BNT162b2 pfizer in SARS-coV-2 igG antibodies titers. Vaccines. (2022) 10:392. doi: 10.3390/vaccines10030392 35335024 PMC8948699

[18] Cuschieri S. The STROBE guidelines. Saudi J Anaesth. (2019) 13:31. doi: 10.4103/sja.SJA_543_18 PMC6398292 30930717

[19] von Elm E Altman DG Egger M Pocock SJ Gøtzsche PC Vandenbroucke JP. The Strengthening the Reporting of Observational Studies in Epidemiology (STROBE) statement: guidelines for reporting observational studies. J Clin Epidemiol. (2008) 61:344–9. doi: 10.1016/j.jclinepi.2007.11.008 18313558

[20] DiaSorin. LIASON SARS-CoV-2 S1/S2 IgG. The fully automated serology test for the detection of SARS-CoV-2 IgG Antibodies (2020). Available online at: https://www.diasorin.com/sites/default/files/allegati/liaisonr_sars-cov-2_s1s2_igg_brochure.pdf.pdf.

[21] Bonelli F Sarasini A Zierold C Calleri M Bonetti A Vismara C. Clinical and analytical performance of an automated serological test that identifies S1/S2-neutralizing igG in COVID-19 patients semiquantitatively. J Clin Microbiol. (2020) 58:e01224–20. doi: 10.1128/JCM.01224-20 PMC7448652 32580948

[22] Romero-Ibarguengoitia ME Rivera-Salinas D Sarti R Levi R Mollura M Garza-Silva A. Efficacy of six different SARS-coV-2 vaccines during a six-month follow-up and five COVID-19 waves in Brazil and Mexico. Vaccines. (2023) 11:842. doi: 10.3390/vaccines11040842 37112754 PMC10142281

[23] Levi R Azzolini E Pozzi C Ubaldi L Lagioia M Mantovani A. One dose of SARS-CoV-2 vaccine exponentially increases antibodies in individuals who have recovered from symptomatic COVID-19. J Clin Invest. (2021) 131:e149154. doi: 10.1172/JCI149154 33956667 PMC8203458

[24] Levi R Ubaldi L Pozzi C Angelotti G Sandri MT Azzolini E. The antibody response to SARS-CoV-2 infection persists over at least 8 months in symptomatic patients. Commun Med. (2021) 1:32. doi: 10.1038/s43856-021-00032-0 35072166 PMC8767777

[25] Ruenkham A Uitrakul S Oberdorfer P Okonogi S Katip W. Comparative safety and effectiveness of heterologous coronaVac–chAdOx1 versus homologous coronaVac vaccination in a real-world setting: A retrospective cohort study. Vaccines. (2023) 11:1458. doi: 10.3390/vaccines11091458 37766134 PMC10535109

[26] Costa Clemens SA Weckx L Clemens R Almeida Mendes AV Ramos Souza A Silveira MBV. Heterologous versus homologous COVID-19 booster vaccination in previous recipients of two doses of CoronaVac COVID-19 vaccine in Brazil (RHH-001): a phase 4, non-inferiority, single blind, randomised study. Lancet. (2022) 399:521–9. doi: 10.1016/S0140-6736(22)00094-0 PMC8782575 35074136

[27] Cheng H Peng Z Si S Alifu X Zhou H Chi P. Immunogenicity and safety of homologous and heterologous prime–boost immunization with COVID-19 vaccine: systematic review and meta-analysis. Vaccines. (2022) 10:798. doi: 10.3390/vaccines10050798 35632554 PMC9142990

[28] Pascuale CA Varese A Ojeda DS Pasinovich ME Lopez L Rossi AH. Immunogenicity and reactogenicity of heterologous immunization against SARS CoV-2 using Sputnik V, ChAdOx1-S, BBIBP-CorV, Ad5-nCoV, and mRNA-1273. Cell Rep Med. (2022) 3:100706. doi: 10.1016/j.xcrm.2022.100706 35926505 PMC9346506

[29] Soltani S Matin BK Gouya MM Zahraei SM Moradi G Chehri O. A prospective cohort study protocol: monitoring and surveillance of adverse events following heterologous booster doses of Oxford AstraZeneca COVID-19 vaccine in previous recipients of two doses of Sinopharm or Sputnik V vaccines in Iran. BMC Public Health. (2023) 23:1415. doi: 10.1186/s12889-023-16265-8 37488541 PMC10364349

[30] Romero-Ibarguengoitia ME González-Cantú A Pozzi C Levi R Mollura M Sarti R. Analysis of immunization time, amplitude, and adverse events of seven different vaccines against SARS-CoV-2 across four different countries. Front Immunol. (2022) 13:894277. doi: 10.3389/fimmu.2022.894277 35967368 PMC9367469

[31] Zember S Bodulić K Balent NC Mikulić R Markotić A Đaković Rode O. Slower waning of anti-SARS-coV-2 igG levels six months after the booster dose compared to primary vaccination. Vaccines. (2022) 10:1813. doi: 10.3390/vaccines10111813 36366322 PMC9698173

[32] Lim SY Kim JY Jung J Yun SC Kim SH. Waning of humoral immunity depending on the types of COVID-19 vaccine. Infect Dis. (2023) 55:216–20. doi: 10.1080/23744235.2023.2165707 36625442

[33] Cagla Karakoc Z. Antibody response to COVID-19 vaccines in healthcare workers: which one is more successful? Homologous or heterologous? Sisli Etfal. (2023) 57(2):216–23. doi: 10.14744/SEMB.2023.48264 PMC10600628 37899804

[34] Shapiro LC Thakkar A Campbell ST Forest SK Pradhan K Gonzalez-Lugo JD. Efficacy of booster doses in augmenting waning immune responses to COVID-19 vaccine in patients with cancer. Cancer Cell. (2022) 40:3–5. doi: 10.1016/j.ccell.2021.11.006 34838186 PMC8595142

[35] Matsumura T Takano T Takahashi Y. Immune responses related to the immunogenicity and reactogenicity of COVID-19 mRNA vaccines. Int Immunol. (2023) 35:213–20. doi: 10.1093/intimm/dxac064 36566501

[36] Pormohammad A Zarei M Ghorbani S Mohammadi M Razizadeh MH Turner DL. Efficacy and safety of COVID-19 vaccines: A systematic review and meta-analysis of randomized clinical trials. Vaccines. (2021) 9:467. doi: 10.3390/vaccines9050467 34066475 PMC8148145

[37] Angkasekwinai N Niyomnaitham S Sewatanon J Phumiamorn S Sukapirom K Senawong S. The immunogenicity and reactogenicity of four COVID-19 booster vaccinations against SARS-CoV-2 variants of concerns (Delta, Beta, and Omicron) following CoronaVac or ChAdOx1 nCoV-19 primary series. Asian Pac J Allergy Immunol. (2021). doi: 10.1101/2021.11.29.21266947 37466962

[38] Li P Wang W Tao Y Tan X Li Y Mao Y. Immunogenicity and reactogenicity of heterologous immunization schedules with COVID-19 vaccines: a systematic review and network meta-analysis. Chin Med J. (2023) 136:24–33. doi: 10.1097/CM9.0000000000002567 36723872 PMC10106236

[39] Rashedi R Samieefar N Masoumi N Mohseni S Rezaei N. COVID-19 vaccines mix-and-match: The concept, the efficacy and the doubts. J Med Virol. (2022) 94:1294–9. doi: 10.1002/jmv.27463 PMC8661746 34796525

[40] Vanaparthy R Mohan G Deepa V Paavani A. Review of COVID-19 viral vector-based vaccines and COVID-19 variants. Infez Med. (2021) 29:328–38. doi: 10.53854/liim-2903-3 PMC8805485 35146337

[41] Mascellino MT Di Timoteo F De Angelis M Oliva A. Overview of the main anti-SARS-coV-2 vaccines: mechanism of action, efficacy and safety. IDR. (2021) 14:3459–76. doi: 10.2147/IDR.S315727 PMC8418359 34511939

[42] Abu Jabal K Ben-Amram H Beiruti K Batheesh Y Sussan C Zarka S. Impact of age, ethnicity, sex and prior infection status on immunogenicity following a single dose of the BNT162b2 mRNA COVID-19 vaccine: real-world evidence from healthcare workers, Israel, December 2020 to January 2021. Eurosurveillance. (2021) 26:1–5. doi: 10.2807/1560-7917.ES.2021.26.6.2100096 PMC7879501 33573712

[43] Silva-Cayetano A Foster WS Innocentin S Belij-Rammerstorfer S Spencer AJ Burton OT. A booster dose enhances immunogenicity of the COVID-19 vaccine candidate ChAdOx1 nCoV-19 in aged mice. Immunology. (2020) 2(3):243–62. doi: 10.1101/2020.10.27.357426 PMC7833318 33521747

[44] He Y Zhang F Liu Y Xiong Z Zheng S Liu W. Clinical characteristics of mild patients with breakthrough infection of omicron variant in China after relaxing the dynamic zero COVID-19 policy. Vaccines. (2023) 11:968. doi: 10.3390/vaccines11050968 37243072 PMC10224174

[45] Deng J Ma Y Liu Q Du M Liu M Liu J. Comparison of the effectiveness and safety of heterologous booster doses with homologous booster doses for SARS-coV-2 vaccines: A systematic review and meta-analysis. IJERPH. (2022) 19:10752. doi: 10.3390/ijerph191710752 36078466 PMC9517782

[46] Riefolo F Castillo-Cano B Martín-Pérez M Messina D Elbers R Brink-Kwakkel D. Effectiveness of homologous/heterologous booster COVID-19 vaccination schedules against severe illness in general population and clinical subgroups in three European countries. Vaccine. (2023) 41:7007–18. doi: 10.1016/j.vaccine.2023.10.011 37858451

